# MDR Gene Expression Analysis of Six Drug-Resistant Ovarian Cancer Cell Lines

**DOI:** 10.1155/2013/241763

**Published:** 2012-12-26

**Authors:** Radosław Januchowski, Karolina Wojtowicz, Patrycja Sujka-Kordowska, Małgorzata Andrzejewska, Maciej Zabel

**Affiliations:** ^1^Department of Histology and Embryology, Poznań University of Medical Sciences, 61-781 Poznań, Poland; ^2^Department of Histology and Embryology, Wrocław Medical University, 50-368 Wrocław, Poland

## Abstract

Ovarian cancer is the leading cause of death among gynaecological malignancies. Multiple drug resistance makes cancer cells insensitive to chemotherapy. In this study, we developed six primary ovarian cancer cell lines (W1MR, W1CR, W1DR, W1VR, W1TR, and W1PR) resistant to drugs such as methotrexate, cisplatin, doxorubicin, vincristine, topotecan, and paclitaxel. A chemosensitivity assay MTT test was performed to assess drug cross-resistance. Quantitative real-time polymerase chain reaction and Western blot were also performed to determine mRNA and protein expression of genes involved in chemoresistance. We observed high cross-resistance to doxorubicin, vincristine, and paclitaxel in the cell lines resistant to these agents. We also found a significant correlation between resistance to these drugs and increased expression of P-gp. Two different mechanisms of topotecan resistance were observed in the W1TR and W1PR cell lines. We did not observe any correlation between MRP2 transcript and protein levels. Cell lines resistant to agents used in ovarian cancer treatment remained sensitive to methotrexate. The main mechanisms of drug resistance were due to P-gp expression in the doxorubicin, vincristine, and paclitaxel resistant cell lines and BCRP expression in the topotecan resistant cell line.

## 1. Introduction

Ovarian cancer represents the most common cause of death among gynaecological malignancies in Europe and North America. The average 5-year survival rate is approximately 40%; however, patients with advanced disease (stages III and IV) have a significantly lower survival rate of only 10%–20% [1]. A high percentage of mortality results from low diagnosis. Most ovarian cancers are diagnosed when the disease has progressed to the advanced stages III or IV, according to the FIGO classification.

Regardless of the stage of the disease, the first line of treatment includes a combined chemotherapeutic regimen of platinum and taxane [2]. Unfortunately, approximately 80% of patients with advanced ovarian cancer who respond well after obtaining the first line of treatment will still have a recurrence and will require continuation of treatment. The second line of chemotherapy usually includes taxane derivatives as well as cisplatin, topotecan, doxorubicin, and gemcitabine [3–7]. The 15%–35% of patients' response to most drugs introduced as a second-line of chemotherapeutic regimen.

Drug resistance is the main cause of ineffective chemotherapy in patients. Several data have been published regarding various cellular mechanisms of drug resistance [8–10]. The most significant and prevalent mechanism of drug resistance is provided by the ability of cancer cells to actively expel the therapeutic agents from the cell via transport proteins. This form of drug resistance is called multiple drug resistance (MDR). MDR development occurs when cancer cells become insensitive to not only the primary cytostatic drug used but also to other pharmaceutical agents that bear no chemical similarities to the structure of cytostatic drugs. Many types of cancers show significant primary resistance to cytostatics, while others acquire characteristics of MDR during chemotherapy. The development of MDR leads to the ineffectiveness of the agent, thereby preventing its further use for treatment [11]. The proteins most implicated in this process belong to the ABC family. These transmembrane proteins use the energy from the hydrolysis of ATP to actively remove drugs from the cell [12]. The critical ABC protein glycoprotein P (P-gp) is encoded by the ABCB1 (multidrug resistance protein 1-MDR1) gene [12, 13]. As expected, P-gp expression is the highest in tumours derived from tissues that normally express P-gp. However, in many other tumours, the expression of P-gp is induced by chemotherapy. This protein is able to actively remove approximately 20 cytostatic drugs from the cell, including [11] paclitaxel [14], doxorubicin [15], and vincristine [16].

Another gene responsible for MDR is MRP1 (MDR-related protein 1; ABCC1). This gene was first described in the non-P-gp MDR small-cell lung carcinoma cell line [12, 17]. MRP1 and P-gp have great similarities in both structure and drug resistance, with the exception of taxanes, which are poor substrates for MRP1. The second member of the multidrug resistance protein (MRP; ABCC) family, MRP2, which is also designed as the canalicular multiorganic anion transporter (CMOAT), is involved in bilirubin glucuronide transport and confers resistance to MRP1 substrates and cisplatin [18]. The role of this protein in the resistance of ovarian cancer to cisplatin has been described in several studies [19, 20]. Another important MDR protein, breast cancer resistance protein BCRP (ABCG2), was cloned from a mitoxantrone-resistant subline of the breast cancer cell line MCF-7 [21]. BCRP lends resistance to many cytostatics, including mitoxantrone and topotecan [12, 22]. Its role in the resistance of ovarian cancer to topotecan is well described [23]. LRP/MVP lung resistance-related protein/major vault protein is an example of a protein involved in MDR that does not belong to the ABC family [24]. It has been reported that LRP expression is correlated with *in vitro* resistance to anticancer drugs such as etoposide, doxorubicin, paclitaxel, and cisplatin [25, 26]. LRP overexpression predicts a poor response to chemotherapy in acute myeloid leukaemia and ovarian carcinoma [12]. 

The current knowledge on the development of drug resistance is based largely on research on drug sensitive/resistant cell lines. During the last few decades, many such cell lines were developed [27, 28]. However, the research was usually limited to 1 or 2 cell resistant lines. In this study, we developed six drug-resistant cell lines from one parental ovarian cancer cell line. We used drugs commonly used for first-line ovarian cancer treatment (paclitaxel and cisplatin), drugs used for the second line of chemotherapeutic treatment (topotecan and doxorubicin), and drugs that are generally not standard for the treatment of ovarian cancer (methotrexate and vincristine). The objective of our research was to compare the development of drug resistance to cytostatics from the first and the second lines of chemotherapy treatment because they have different mechanisms of action. Additionally, the goal of our study was to compare the cross-resistance between cell lines developed in the presence of these drugs, examine the expression of five genes responsible for the development of MDR, and determine a correlation between the establishments of drug resistance to any treatment with the expression of MDR genes. 

## 2. Materials and Methods

### 2.1. Reagents and Antibodies

Methotrexate, cisplatin, doxorubicin, vincristine, topotecan, and paclitaxel were obtained from Sigma (St. Louis, MO). RPMI-1640 medium, foetal bovine serum, antibiotic-antimycotic solution, and L-glutamine were also purchased from Sigma (St. Louis, MO). A Cell Proliferation Kit I (MTT) was purchased from Roche Diagnostics GmbH (Mannheim, Germany). Mouse anti-MRP1 monoclonal antibody (Ab) (IU2H10), goat anti-MRP2 polyclonal (Ab) (H-17), rabbit anti-ABCG2 polyclonal Ab (H-70), rabbit anti-GADPH polyclonal Ab (FL-335), donkey anti-goat horseradish peroxidase (HRP)-conjugated Ab, goat anti-mouse HRP-conjugated Ab, and goat anti-rabbit HRP-conjugated Ab were purchased from Santa Cruz Biotechnology (Santa Cruz, CA). Mouse monoclonal anti-P-glycoprotein Ab (C219) and mouse monoclonal anti-MVP/LRP Ab (MVP 37) were obtained from Alexis Biochemicals (Lörrach, Germany). 

### 2.2. Cell Lines and Cell Culture

The primary human ovarian cancer cell line W1 was established in our department (December 2009) from tissue obtained from an untreated patient diagnosed with ovarian cancer. Sublines resistant to methotrexate (W1MR; W1 methotrexate resistant), cisplatin (W1CR; W1 cisplatin resistant), doxorubicin (W1DR; W1 doxorubicin resistant), vincristine (W1VR; W1 vincristine resistant), topotecan (W1TR; W1 topotecan resistant), and paclitaxel (W1PR; W1 paclitaxel resistant) were generated by exposure of the W1 line to incremental increasing concentrations of each drug. The cells were seeding in the concentration of 10 thousand cells/cm^2^ in 25 cm^2^ flask. Media were supplemented with relevant drug. Initial drugs exposure were at a concentration of MTX 2 ng/mL, CIS 20 ng/mL, Dox 10 ng/mL, Vin 0,5 ng/mL, TOP 0,5 ng/mL, and PAC 1 ng/mL with the cell line exposed three times for 3-day periods during a 3–6-week period allowing for growth recovery between cycles. After the completion of three cycles of drug, the dose was doubled, and the procedure was repeated until the final drug levels were achieved. The final concentration of each drug was MTX 28 ng/mL, CIS 1000 ng/mL, DOX 100 ng/mL, WIN 10 ng/mL, TOP 24 ng/mL, and PAC 1100 ng/mL according to Dietel et al. (1993) [29]. These consternations were twofold greater than the concentration in the plasma 2 hours after intravenous administration [29]. All cell lines were maintained as a monolayer in complete medium [RPMI 1640 medium supplemented with 10% (v/v) foetal bovine serum, 2 pM L-glutamine, penicillin (100 units/mL), streptomycin (100 units/mL) and amphotericin B (25 *μ*g/mL)] at 37°C in a 5% CO_2_ atmosphere.

### 2.3. Drug Sensitivity Assay

The drug sensitivity of the W1 cell line and the drug resistant cell lines were confirmed by the MTT cell survival assay. Briefly, all cell lines were seeded at a density of 5,000 cells/well in 96-well plates. The cells were allowed to grow for 48 hours and subsequently treated with fresh medium supplemented with or without increasing concentrations of drugs and incubated for 72 h at 37°C. After 72 h of exposure, 10 *μ*L of the MTT labelling reagent was added to the medium (the final concentration of MTT was 0.5 mg/mL), and the cells were incubated for an additional 4 h. Following this process, 100 *μ*L of solubilisation solution was added to each well. The absorbance of each sample was measured in a microplate reader at 570 nm with a reference wavelength of 720 nm, according to the manufacturer's protocol. The negative control was conducted using cell-free culture medium containing both the MTT reagent and solubilisation solution. The experiments were repeated three times, and each concentration in a given experiment was tested in duplicates. Cell viability was expressed as a percentage of the untreated control (means ± SEM).

### 2.4. Examination of ABCB1 Gene Expression by Using Q-PCR

Changes in ABCB1, ABCC1, ABCC2, ABCG2 and LRP gene expression in the W1 and drug-resistant cell lines were examined. RNA was isolated using the Gene Matrix Universal RNA purification Kit (EURx Ltd.), as described by the manufacturer's protocol. Reverse transcription was performed using the M-MLV reverse transcriptase (Invitrogen) as described in the manufacturer's protocol using a thermal cycler (Veriti 96 well Thermal Cycler). 2 *μ*g of RNA was used to cDNA synthesis. Real-time PCR was performed using the Eppendorf PCR System (Mastercycler realplex^4^), Maxima SYBR Green/ROX qPCR Master Mix (Fermentas) and sequence-specific primers, as indicated in Table 1. Glyceraldehyde-3-phosphate dehydrogenase (GADPH), *β*-actin, hypoxanthine-guanine phosphoribosyltransferase 1 (HRPT1) and beta-2-microglobulin (*β*2M) served as the normalising genes (geometric mean) against which changes in the examined genes expression were compared. Gene expression was analysed using the relative quantification (RQ) method. RQ estimates the difference at the level of gene expression against a calibrator (W1 drug sensitive line) (RQ of the calibrator = 1). The W1 cell line was used as the calibrator. The analysis was conducted employing the standard formula: RQ = 2^−ΔΔCt^ (where ΔΔCt = ΔCt for the sample (drug-resistant line) −ΔCt for the calibrator (drug sensitive line)). The graphs were made using Sigma Plot.

For amplification, 12,5 *μ*L of Maxima SYBR Green/ROX qPCR Master Mix (Fermentas), 1 *μ*L of each primer (Oligo, Warsaw, Poland) (Table 1), 9,5 *μ*L of water, and 1 *μ*L of cDNA solution were mixed together. One RNA sample of each preparation was processed without RT-reaction to provide a negative control in subsequent PCR. Sample amplification included a hot start (95°C, 15 minutes) followed by 50 cycles of denaturation at 95°C for 15 seconds, annealing at 60°C for 30 seconds, and extension at 72°C for 30 seconds. After amplification, Melt Curve analysis was performed to analyze product melting temperature. The amplification products were also resolved by 3% agarose gel electrophoresis and visualized by ethidium bromide staining.

### 2.5. SDS-PAGE and Western Blot Analysis of P-gp, MRP1, MRP2, BCRP, LRP

Cells (1 × 10^6^ cells/50 *μ*L lysis buffer) were lysed in buffer containing 20 mM Tris-HCl (pH 7.5), 100 mM NaCl, 5 mM EDTA, Protein Inhibitor Cocktail (ROCHE) and 1% Triton X-100 for 60 min at 4°C. The lysates were centrifuged at 12000 ×g for 15 min at 4°C, and protein concentration was determined using the Bio-Rad (Hercules, CA) protein assay system. Thirty micrograms of the protein was resuspended in a solution of 40 *μ*L of 200 mM Tris-HCl (pH 6.8), 5% SDS, 10% glycerol, 0.25% 2-mercaptoethanol, and 0.1% bromophenol blue. The resuspended protein was loaded into each well and separated on a 7% Tris-glycine gel using the SDS-PAGE technique. The proteins were transferred to a PVDF membrane and blocked with 5% milk in TBS/Tween (0.1 M Tris-HCl, 0.15 M NaCl, 0.1% Tween 20), followed by immunodetection with either mouse anti-P-gp Ab (C219) at 1 : 500 dilution, mouse anti-MRP1 Ab (IU2H10) at 1 : 500 dilution, goat anti-MRP2 Ab (H-17) at 1 : 500 dilution, rabbit anti-ABCG2 Ab (H-70) at 1 : 500 dilution, or mouse anti-MVP/LRP (MVP37) Ab at 1 : 500 dilution with the appropriate HRP-conjugated secondary Ab. Chemiluminescence detection of the bands was performed using the enhanced chemical luminescence (ECL) kit and Hyperfilm ECL from Amersham (Piscataway, NJ). The Western blot was quantified by densitometry analysis of the band intensity in the autoradiogram using the GelDoc-It Imaging System and the Vision WorkLS software. To normalise protein loading of the lanes, the membranes were stripped and reblotted with rabbit anti-GADPH Ab (FL-335) at 1 : 500 dilution, donkey anti-goat HRP-conjugated Ab, and goat anti-rabbit HRP-conjugated Ab.

## 3. Results

### 3.1. Characteristics of W1 and W1 Sublines

**Figure 1:**
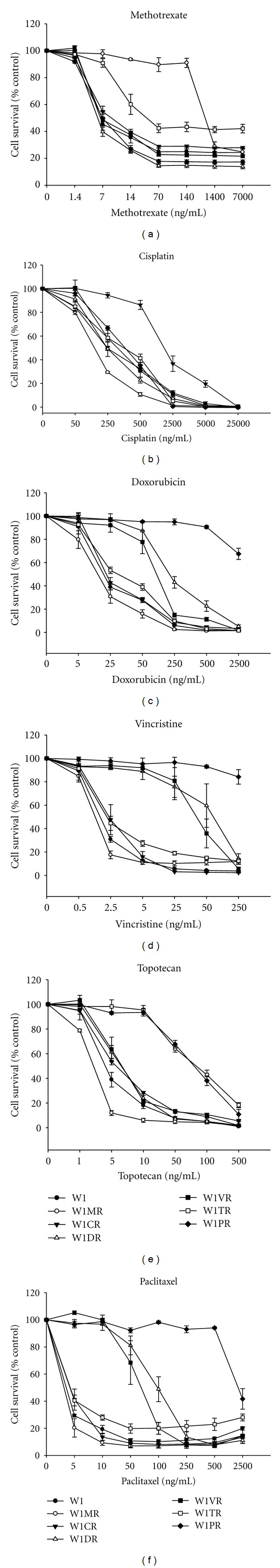
MTT cell survival assay. W1, W1MR, W1CR, W1DR, W1VR, W1TR, and W1PR cells were seeded at density of 5000 cells/well in 96-well plates and treated with or without increasing concentration of methotrexate (a), cisplatin (b), doxorubicin (c), vincristine (d), topotecan (e), and paclitaxel (f), at 37°C for 72 h, and viability of cells was determined. The experiments were repeated three times, and each concentration was tested in triplicate in each experiment. Viability was expressed as a percent on an untreated control (mean ± SEM).

The W1MR, W1CR, W1DR, W1VR, W1TR, and W1PR drug-resistant variant sublines of the W1 human ovarian cancer line were all established by the stepwise selection of W1 cells cultured in growth media with increasing drug concentrations. To determine the sensitivity of the W1 and drug-resistant W1 sublines to methotrexate, cisplatin, doxorubicin, vincristine, topotecan, and paclitaxel, cells were treated with different concentrations of each drug for 72 h. The dose-dependent effect of methotrexate on W1 and the drugs-resistant cell lines were observed (Figure 1(a) and Table 2). The W1, W1CR, W1DR, W1VR, and W1PR cell lines were all sensitive to methotrexate treatment. At this concentration (14 ng/mL), the W1MR cell line was not sensitive, while the W1TR cell line displayed partial resistance to methotrexate treatment. 

The response of cell lines to cisplatin treatment was also observed (Figure 1(b)). Compared to the other lines, the W1CR cell line was more resistant to cisplatin. The IC50 analysis (Table 2) showed statistically significant changes in W1PR and W1MR cell lines, where an increase and decrease of resistance, respectively, were conferred relative to the W1 cell line.

The effect of doxorubicin, vincristine, and paclitaxel on the cell lines was investigated (Figures 1(c), 1(d), and 1(f)). We observed a high cross-resistance between the W1DR, W1VR, and W1PR cell lines to doxorubicin, vincristine, and paclitaxel drugs. The W1DR and W1VR cell lines shared similar levels of resistance to doxorubicin, vincristine, and paclitaxel. In contrast, the W1PR cell line appeared more resistant to doxorubicin and vincristine than the actual lines developed in the presence of doxorubicin and vincristine (Table 2). 

The cell lines W1TR and W1PR were resistant to topotecan and shared a similar dose-dependent response profile (Figure 1(e)). Other cell lines were also topotecan sensitive, but the W1MR cell line exhibited the greatest sensitivity (Table 2).

### 3.2. Gene Expression Analysis in Drug-Resistant Ovarian Cancer Cell Lines

**Figure 2:**
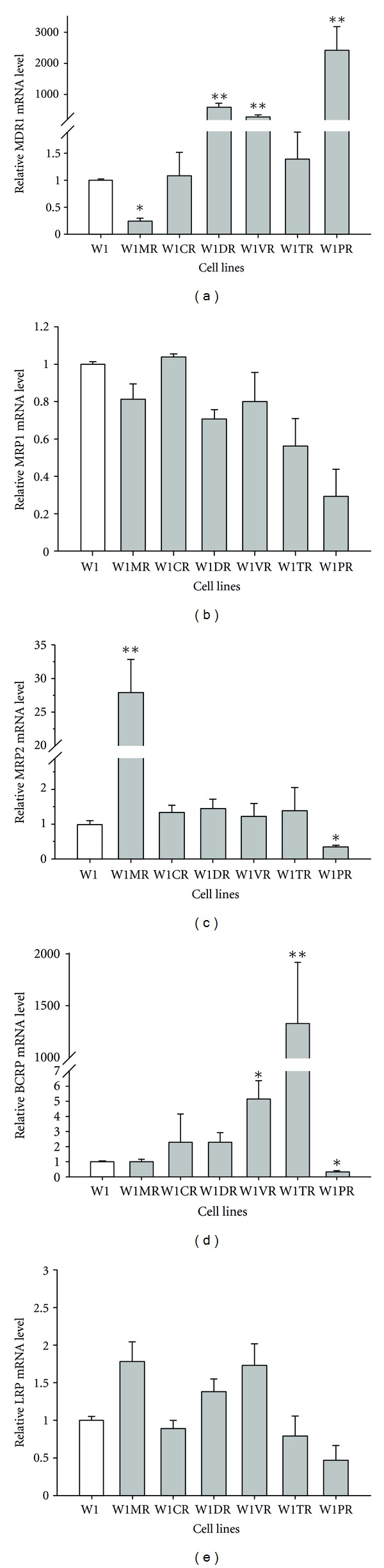
Expression analysis (Q-PCR) of MDR1 (a), MRP1 (b), MRP2 (c), BCRP (d), and LRP (e) genes. The figure presents relative gene expression in resistant cell lines (grey bars) with respect to the W1 cell line (white bars) assigned as 1. Values were considered significant at *P < 0.01 and **P < 0.001.

To determine whether the development of drug-resistance in the variant sublines of the W1 parental line is associated with increased expression of genes involved in MDR, expression of the following mRNA levels was assessed: MDR1, MRP1, MRP2, BCRP, and LRP. We did not observe statistically significant changes in the MRP1 or LRP transcript levels between the cell lines (Figures 2(b) and 2(e)).

The transcript level of MDR1 significantly increased in the doxorubicin, vincristine, and paclitaxel (W1DR, W1VR and W1PR) resistant cell lines (*P* < 0.001). In contrast, the MDR1 transcript level significantly decreased in the methotrexate-resistant cell line (Figure 2(a)) (*P* < 0.01).

The MRP2 transcript level was significantly higher in the methotrexate-resistant cell line (*P* < 0.001) and significantly lower in the paclitaxel-resistant cell line (*P* < 0.01) (Figure 2(c)). 

BCRP expression increased in the vincristine (*P* < 0.01) and topotecan (*P* < 0.001) resistant cell lines. However, expression of BCRP was variable in these two cell lines. We observed approximately sixfold higher transcript levels in the W1VR cells, and expression in the W1TR cells increased by a factor of more than 1,000. In contrast, the expression of BCRP in the W1PR cells significantly decreased (*P* < 0.01) (Figure 1(d)). 

### 3.3. Western Blot Analysis

**Figure 3:**
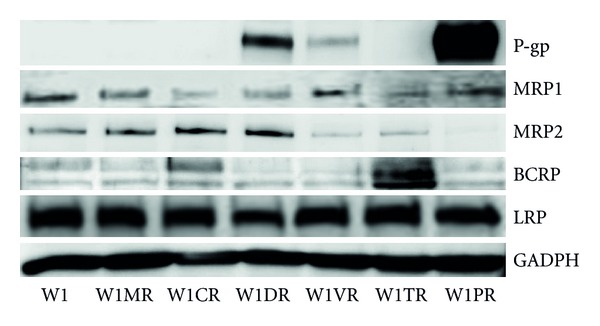
P-gp, MRP1, MRP2, BCRP, and LRP protein expression in W1 and drug-resistant cell lines. The cellular proteins were separated using 7% page and transferred to PVDF, and the membrane was immunoblotted with either primary Ab or HRP-conjugated secondary Ab.

Western blot analysis (Figure 3) of the P-gp and BCRP proteins validated the transcript expression results. We observed increased expression of P-gp protein in the cell line resistant to paclitaxel, pronounced expression in the cell lines resistant to doxorubicin and some expression in the cell line resistant to vincristine; we observed a very high correlation between transcript and protein levels. We observed an increased expression of the BCRP protein in the topotecan resistant cells. In contrast, the protein levels of MRP2 did not correlate with its transcript levels. We found increased MRP2 expression in the W1MR cell line; however, the expression was higher in the W1CR and W1DR cell lines. Expression of MRP2 in the W1VR and W1TR cell lines was lower than in control, while it was barely detectable in the W1PR line. Expression of MRP1 in W1CR, W1DR, and W1TR was lower than that in control. We observed very stable level of LRP protein in all investigated cell lines.

### 3.4. Correlation between Chemosensitivity and Gene Expression in the Cell Lines

To assess whether expression of MDR genes was correlated with resistance to a specific drug treatment, correlation analyses of MDR1, MRP2, and BCRP with IC50 levels for methotrexate, cisplatin, doxorubicin, vincristine, topotecan, and paclitaxel were performed. We observed a high degree of correlation between the MDR1 transcript and protein and resistance to doxorubicin, vincristine and paclitaxel (Tables 3 and 4). Similarly, a high correlation was observed between resistance to methotrexate and MRP2 transcript level (Table 3). However, we did not find any correlation between the MRP2 protein level and IC50 for any of the drugs in our study (Table 4). In spite of the high transcript and protein levels of BCRP in the W1TR cell line, we did not observe a correlation between BCRP expression and resistance to topotecan treatment (Tables 3 and 4). 

## 4. Discussion

In our study, we compared development of multiple drug resistance to the parental W1 ovarian cancer cell line in response to cytostatic agents used in ovarian cancer chemotherapy, all of which have different mechanisms of action. The drug cross-reactivity study showed that the parental W1 cell line was sensitive to all investigated drugs with IC50 below their therapeutic concentration [29]. Comparisons between our drug-resistant cell lines, which were generated from drugs that are commonly used as chemotherapy to treat ovarian cancer, revealed that only the W1TR cell line showed partial cross-resistance to methotrexate. The remaining cell lines were methotrexate sensitive. Importantly, because our results showed that cell lines resistant to drugs used in the first and the second line of ovarian cancer treatment remained sensitive to methotrexate, it can be considered a suitable alternative agent for the treatment of ovarian cancer. Of course these results should be confirmed on established ovarian cancer cell lines. 

The drug most commonly prescribed in ovarian cancer treatment is cisplatin [30]. In our results, we did not observe any cross-resistance between the W1CR line and other cell lines resistant to cisplatin. The lack of cross-resistance between cisplatin and other drugs used in ovarian cancer chemotherapy validates the use of cisplatin as a first-line chemotherapeutic agent. 

A similar pattern of cross-resistance to doxorubicin, vincristine, and paclitaxel has been observed in the investigated cell lines. The cell lines studied here can be divided in two groups: sensitive to the tested drugs (W1, W1MR, W1CR, and W1TR) and those resistant to the drugs (W1DR, W1VR, and W1PR). These results may have been expected because cross-resistance between cell lines resistant to these drugs has frequently been documented in the literature [31–33]. Furthermore, cross-resistance between the W1DR and W1PR cell lines suggest that doxorubicin is not a recommended second-line cytostatic drug in patients who have developed resistance to paclitaxel as first-line chemotherapy. 

The W1PR line has shown a similar pattern of response to the topotecan like topotecan-resistant cell line, W1TR; however, the W1TR line showed no resistance to paclitaxel. Here, we observed only a one-sided cross-resistance, suggesting that topotecan may not serve as a good cytostatic for ovarian cancer treatment because resistance to paclitaxel has already developed. Therefore, topotecan might be better applied as a drug for first-line chemotherapy. The best recognised protein responsible for MDR is P-gp, encoded by the ABCB1 (MDR1) gene. We observed high levels of the transcript and the protein in cell lines resistant to doxorubicin, vincristine and paclitaxel. This observation is consistent with published data showing that P-gp has broad substrate specificity, including natural products such as anthracyclines, vinca alkaloids, and taxanes [8, 12–16, 34]. Both the transcript and protein levels of P-gp exhibited a strong correlation with IC50 for doxorubicin, vincristine, and paclitaxel in W1DR, W1VR, and W1PR cell lines. This result suggests that P-gp played a critical role in resistance to these drugs in the investigated cell lines. 

The lack of correlation between the transcript and protein levels of MRP2 may result from increased transcription or increased transcript stability caused by methotrexate treatment. It is also possible that there exists another so far not described MRP2 isoform, and the difference between the transcript and protein levels may reflect different MRP2 isoforms. Regardless, we observed a pronounced correlation between the MRP2 transcript level and methotrexate resistance in our cell lines. This correlation suggests that MRP2 plays a critical role in methotrexate resistance in the investigated cell lines, which is also consistent with several previously published data showing a correlation between MRP2 expression and methotrexate resistance [35, 36]. High MRP2 expression has been observed in cell lines resistant to cisplatin [19, 37]. In our research, we did not observe an increase in the MRP2 transcript or protein level in the cisplatin resistant cell line, which may be due to the fact that MRP2 protein is not the only protein responsible for cisplatin resistance. Glutathione [38], glutathione metabolising enzymes [39], and metallothioneins [40] are also responsible for resistance to cisplatin. The differences in the transcript and protein levels of MRP2 and the role of MRP2 in the resistance of the investigated cell lines require further investigation.

High BCRP transcript and protein levels in the topotecan resistant cell line have been well established [23, 41, 42] and have been subsequently confirmed by our results. Increased BCRP transcript in the W1VR cell line was not expected because vincristine is not a substrate of BCRP. However, BCRP expression increased only sixfold in comparison to over a thousandfold increase in the W1TR line, for which BCRP protein level was not altered. The topotecan resistant cell line was shown to be partially resistant to methotrexate; this resistance may be a direct result of high levels of BCRP expression because methotrexate is a substrate for BCRP. We have observed two different mechanisms of topotecan resistance. The W1TR cell line developed the “classical” topotecan resistance, based on increased BCRP expression [23, 41, 42]. Additionally, we have observed a similar dose-dependent response pattern to topotecan in the paclitaxel resistant cell line, in spite of its low BCRP expression, which may be due to the high levels of P-gp expression in the W1PR cell line. Published reports have suggested that high levels of P-gp expression play a significant role in topotecan resistance [43]. However, two other cell lines with high expression of P-gp, W1DR and W1VR, demonstrated a sensitivity to topotecan. Accordingly, how much of a role MDR1 plays in topotecan resistance and whether other proteins are involved in this drug resistance mechanism must be evaluated. For example, it has been shown that MRP4 plays a role in topotecan resistance [44]. Therefore, the resistance of the W1PR cell line to topotecan requires further investigation. 

Contrary to other published results, we observed methotrexate sensitivity in our cell lines expressing high levels of P-gp. According to previously published reports, methotrexate is a substrate for P-gp [45], but our cell lines that have high expression of P-gp (W1DR, W1VR, and W1PR) were all methotrexate sensitive. These seemingly contradictory results may be explained by studies that showed that P-gp is an efficient methotrexate transporter in cells that were deficient in the methotrexate carrier [46, 47]. 

## 5. Conclusions

In summary, some of mechanisms of drug resistance are well known, and our results suggest that it is possible to predict cross-resistance to other drugs when the classical MDR, which is correlated with P-gp expression, is involved. Cases of other resistances such as topotecan, methotrexate, and cisplatin resistance appear to be more complex, and further analyses of MDR development must be explored. Our results confirm that cisplatin is an effective drug for first-line chemotherapy in ovarian cancer treatment. The efficacy of topotecan and doxorubicin as the second lines of chemotherapy may be limited because of their cross-resistance with paclitaxel, which is used as a first line of chemotherapeutic treatment. Therefore, methotrexate may be considered to be an alternative therapy for ovarian cancer treatment because no cross-resistance was observed in our cell lines.

## Figures and Tables

**Table 1:** Oligonucleotide sequences used for Q-PCR analysis.

Transcript	Sequence (5′-3′ direction)	ENST number (http://www.ensembl.org/)	Product size (bp)
ABCB1	TGACAGCTACAGCACGGAAG	00000265724	131 bp
	TCTTCACCTCCAGGCTCAGT		
ABCC1	GAGAGTTCCAAGGTGGATGC	00000399410	149 bp
	AGGGCCCAAAGGTCTTGTAT		
ABCC2	TACCAATCCAAGCCTCTACC	00000370449	104 bp
	AGAATAGGGACAGGAACCAG		
ABCG2	TTCGGCTTGCAACAACTATG	00000237612	128 bp
	TCCAGACACACCACGGATAA		
LRP	TGAGGAGGTTCTGGATTTGG	00000357402	135 bp
	TGCACTGTTACCAGCCACTC		
GADPH	GAAGGTGAAGGTCGGAGTCA	00000229239	199 bp
	GACAAGCTTCCCGTTCTCAG		
*β*-actin	TCTGGCACCACACCTTCTAC	00000331789	169 bp
	GATAGCACAGCCTGGATAGC		
HRPT1	CTGAGGATTTGGAAAGGGTG	00000298556	156 bp
	AATCCAGCAGGTCAGCAAAG		
*β*2M	CGCTACTCTCTCTTTCTGGC	00000558401	133 bp
	ATGTCGGATGGATGAAACCC		

**Table 2:** Summary of cell line cross-resistance to drug treatment.

Cell line	IC50 (ng/mL)					
	Methotrexate	Cisplatin	Doxorubicin	Vincristine	Topotecan	Paclitaxel
	7.02	253	20.8	1.85	4.19	3.54
W1	(5.85–8.58)	(231–270)	(20.5–21.1)	(1.82–1.87)	(3.55–4.95)	(3.47–3.65)
	1	1	1	1	1	1

	970	168	17.0	1.53	2.72	3.19
W1MR	(858–1086)	(161–174)	(12.4–21.5)	(1.42–1.60)	(2.55–2.81)	(2.77–3.73)
	138↑***	0.66↓**	0.82	0.83↓**	0.65↓*	0.90

	9.00	1991	23.3	2.57	5.66	4.43
W1CR	(6.80–8.12)	(1630–2470)	(20.5–24.5)	(1.66–3.53)	(5.07–6.55)	(3.43–5.55)
	1.28	7.87↑***	1.12	1.39	1.35	1.25

	6.00	258	215	106	6.4	109
W1DR	(5.90–6.60)	(213–337)	(165–248)	(30.5–131)	(5.67–7.44)	(76.6–152)
	0.85	1.02	10.3↑***	57.3 ↑**	1.52↑*	30.8↑***

	7.53	320	132	45.3	6.35	64.8
W1VR	(6.10–9.70)	(243–422)	(92–167)	(24.4–65.6)	(4.69–7.74)	(44.6–85.6)
	1.07	1.26	6.35↑***	24.5 ↑***	1.52	18.3↑***

	39	374	30.9	2.34	83.9	4.24
W1TR	(22–67)	(285–451)	(21.6–33.9)	(1.68–3.10)	(70.9–98)	(3.92–4.86)
	5.55↑*	1.48	1.49	1.26	20.0↑***	1.20

	7.53	383	4241	1155	80.2	2268
W1PR	(5.9–8.4)	(366–413)	(3136–5624)	(548–2100)	(72.5–92.8)	(1868–2788)
	1.07	1.51↑**	204↑***	624↑***	19.1↑***	641↑***

IC50 mean is indicated for each drug. The drug resistance in W1 cell line was assigned as 1. Underlined values indicate multiplicities of resistance with respect to W1 cell line. *P < 0.05; **P < 0.01; ***P < 0.001.

**Table 3:** Correlation between transcript level and IC50 for cytostatic drugs (Pearson correlation—*R* ^2^).

Gene	Cytostatic drug					
	Methotrexate	Cisplatin	Doxorubicin	Vincristine	Topotecan	Paclitaxel
MDR1	0.06	0.03	0.96***	0.97***	0.3	0.96***
MRP2	0.99***	0.06	0.05	0.05	0.09	0.05
BCRP	0.02	0.01	0.03	0.04	0.44	0.03

Values were considered significant at ***P < 0.001.

**Table 4:** Correlation between protein levels and IC50 for cytostatic drugs (Pearson correlation—*R* ^2^).

Gene	Cytostatic drug					
	Methotrexate	Cisplatin	Doxorubicin	Vincristine	Topotecan	Paclitaxel
MDR1	0.0549	0.0278	0.9483***	0.9658***	0.3067	0.9486***
MRP2	0.088	0.1529	0.225	0.2388	0.4828	0.254
BCRP	0.0276	0.0019	0.0432	0.0499	0.4059	0.0442

***P < 0.001.
